# Dr. Reid’s Method of Reducing Dislocation of the Femur on the Dorsum Ilii

**Published:** 1851-12

**Authors:** 


### EDITORIAL DEPARTMENT.

*Dr. Reid's method of Reducing Dislocation of the Femur on the Dorsum Ilii.*— The article by Dr. Reid in a former number of this Journal, as was anticipated, has produced somewhat of a sensation. Several results, which were to have been expected, are observable. *First,* the plan proposed by Dr. R., which, should it prove generally successful, would be, as must be universally admitted, a very great improvement in surgery, apparently attracts no attention in some parts of our country. It is not noticed even as a doubtful or fanciful novelty. The reason for this we will not now stop to consider. *Second,* the feasibility of the plan, in the majority of cases, is doubted by some. Time will settle this question. Let the plan be tried sufficiently to determine how far it is available. This is due to the importance of the subject. *Third,* the originality of the plan is denied. This has been done, with the manifestation of considerable feeling, by several writers in the Boston Med. and Surg. Journal. One would imagine, from the tenor of some of these communications, that Dr. Reid had been at the gratuitous pains of telling the medical public only what every practitioner already knew, and what might be found in every text book on surgery. Now practitioners of surgery have been accustomed to reduce dislocation on the dorsum in the mode proposed by Dr. Reid, or they have not. Is there any room for doubt on this point? The merit of the improvement, or the innovation, hinges, not on the question whether Dr Reid is the first to develop and demonstrate the feasibility of the plan, but on the question whether the plan has been hitherto in vogue or not. Dr. Reid does not assert that others may not have conceived the same idea. Indeed, he says he has been informed that the late Prof. Nathan Smith, did teach a plan somewhat similar. But no such plan has ever come into general use, and, if adopted at all, this has been the case to a very limited extent. Do the text books inculcate any such plan? No. And are not the text books the exponents of the established principles of practice? There is a distinction between originality and priority. We do not doubt that Dr. Reid was led to the plan he has proposed, by his own reflections and investigations. We are not prepared to say that others may not have devoted attention to the subject and arrived at similar conclusions. But pulleys and counter extension have continued to be employed in reducing luxations of the femur on the dorsum of the ilium, and reduction by means of them is still taught in the best medical schools and in the most approved surgical works. Dr. Reid's plan is therefore, as he states, entirely opposéd to custom and authority, and should the advantages which he claims for it become fully established by reiterated observations, he will be rightfully considered the originator of a highly important improvement in practical surgery, and, as such, his name will be distinguished in the annals of medicine. A readiness to depreciate praiseworthy exertions in others, and to impute improper or dishonest motives, betokens a pitiful spirit, the exhibition of which should provoke only contempt.

